# Utilization of Ayurveda, Yoga, Naturopathy, Unani, Siddha, and Homoeopathy (AYUSH) Practitioners’ Services Among Older Adults: Results From the Longitudinal Aging Study in India

**DOI:** 10.7759/cureus.62192

**Published:** 2024-06-11

**Authors:** Parimala Mohanty, Jugal Kishore, Geeta C Acharya, Itishree Mohanty, Lipilekha Patnaik, Bratati Bhowmik, Monalisha Sahoo, Nancy Satpathy, Prasanta K Sahoo, Pratap K Jena

**Affiliations:** 1 Public Health, Indian Institute of Technology Guwahati, Guwahati, IND; 2 Community Medicine, Vardhman Mahavir Medical College and Safdarjung Hospital, New Delhi, IND; 3 Community Medicine, Kalinga Institute of Medical Sciences, Bhubaneswar, IND; 4 Hospital Administration, Kalinga Institute of Industrial Technology (KIIT), Bhubaneswar, IND; 5 Community Medicine, Institute of Medical Sciences and SUM Hospital, Siksha 'O' Anusandhan deemed to be University, Bhubaneswar, IND; 6 Public Health, Kalinga Institute of Industrial Technology (KIIT), Bhubaneswar, IND; 7 Public Health, Indian Council of Medical Research (ICMR), New Delhi, IND; 8 Ayurveda, Yoga & Naturopathy, Unani, Siddha, and Homeopathy (AYUSH), All India Institute of Medical Sciences Bhubaneswar, Bhubaneshwar, IND; 9 Healthcare Management, Swiss School of Business and Management (SSBM) Geneva, Geneva, CHE

**Keywords:** healthcare services, indian system of medicine, alternative system of medicine, older adults, ayush

## Abstract

Background: Ayurveda, yoga, naturopathy, Unani, Siddha, and homeopathy (AYUSH) form an alternative system of medicine in India. Understanding the utilization of AYUSH practitioners’ services is crucial to substantiating the current government initiatives to mainstream AYUSH in the Indian health system. The utilization of AYUSH practitioners’ services among different sub-populations, including older adults, for various health conditions is underexplored. The present study explores the utilization of AYUSH practitioners’ service among older Indian adults and its correlates.

Methods: During 2017-2018, the Longitudinal Aging Study in India (LASI) conducted a nationally representative study among adults aged 45 years or more and their spouses. The study leveraged this data from publicly available LASI. Descriptive analysis and cross-tabulation were performed using a subset of older adults (age ≥ 60 years, n = 31,464). The utilization of AYUSH practitioners’ services was taken as the outcome variable. A logistic regression model was employed to understand the independent effect of various explorative variables on the use of AYUSH practitioners' services.

Results: One in 14 older adults utilized the services of AYUSH practitioners. The socio-demographic factors that were looked at, including religion, residence, and caste were significant independent factors for AYUSH consultation. Among chronic conditions, hypertension (use-5.6%, AOR: 1.24, CI: 1.09-1.40), diabetes (use-4.2%, AOR: 1.31, CI: 1.09-1.57), and arthritis (use-9.1%, AOR: 0.59, CI: 0.52-0.67) were independent determinants of AYUSH practitioners' service utilization. In the fully adjusted model, the effect of explanatory variables is almost similar to that in the minimally adjusted model. Only the effect of the female gender was accentuated in magnitude, whereas the effect of diabetes was partially attenuated.

Conclusion: The preference for AYUSH practitioners’ service among older adults is determined by the complex interplay between socio-demographic factors and disease conditions. Though utilization of AYUSH practitioners’ service was high among certain underprivileged sections, it is assuring that education and income do not affect older populations’ preference for AYUSH practitioners' service.

## Introduction

The Indian Systems of Medicine (ISM), comprising Ayurveda, Yoga, Naturopathy, Unani, Siddha, and Homeopathy (AYUSH), have been widely practiced in India, neighboring Asian countries, and some developed nations [1]. These systems are well-known globally for their ability to supplement illness prevention, treatment, and general health maintenance. The ISM was practiced long before the current health system was established [2]. AYUSH offers personalized care, natural remedies, and lifestyle management, crucial for managing chronic conditions and promoting healthy aging. These traditional systems complement conventional medicine, providing cost-effective and holistic solutions for the elderly, emphasizing preventive measures, and enhancing quality of life [2]. Limited government patronage has led to the neglect of the ISM in both pre- and post-independent India. This lack of government support has resulted in a shortage of resources, funding, and attention to the development and promotion of these systems of medicine [3,4].

Experts and health committees in India, including the Mudaliar Committee (1962), have repeatedly advised integrating ISM with modern medicine to promote holistic primary healthcare [5]. In 1995, the Department of ISM and Homeopathy was established to institutionalize ISM. The department was created for the formulation and implementation of policies related to the promotion and development of ISM, as well as for the regulation of education, research, and practice in the field. The department's establishment was the first significant step toward ISM recognition and promotion in India. In 2005, India launched the National Rural Health Mission (NRHM) to enhance the health infrastructure and service delivery in rural areas. As a result of this mission, the government introduced the AYUSH system of medicine into the mainstream healthcare system. By integrating AYUSH systems into public health services, the government aimed to provide a more holistic approach to healthcare and make it more accessible and affordable to people. The integration of AYUSH systems was expected to complement modern medicine and address the healthcare needs of the population more comprehensively [6,7]. The approach of integrating AYUSH systems into mainstream healthcare in India was successful in utilizing AYUSH practitioners to manage community health issues at various levels. This prompted the Department of AYUSH to launch the National AYUSH Mission in 2014, which aimed to provide affordable, sustainable, and accessible healthcare. The formation of the Ministry of AYUSH in November 2014 marked an important milestone in the popularization and strengthening of AYUSH services in India. However, despite governmental patronage, research into AYUSH healthcare and its utilization among community members remains limited. Therefore, more efforts are needed to promote and advance AYUSH healthcare in India [7-12]. The scope of earlier studies on ISM utilization has been limited, as most studies have typically focused on small, specific geographic areas, instead of taking a more comprehensive approach that examines the use of ISM across a wider range of regions and demographic groups [9]. Previous studies on ISM utilization have utilized a limited sample size, potentially rendering the findings unrepresentative of the broader population [13,14]. These limitations result in a lack of evidence to guide the formulation of national policies concerning the use of ISM.

Therefore, a comprehensive investigation of AYUSH service consumption across sub-sections of population, regional, socioeconomic, and demographic categories is necessary to understand its potential for healthcare delivery [7]. As a result, the study aims to explore the utilization of AYUSH practitioners' services among older adults in India. It intends to do so by using a national survey called the Longitudinal Aging Study in India (LASI) conducted in 2017-2018. The study intends to provide crucial insights into the utilization patterns of AYUSH services among various sub-populations and disease conditions. The study's findings could potentially aid in policy formulation and decision-making regarding the integration of AYUSH systems with modern medicine and primary healthcare in India.

## Materials and methods

Study setting and sample

This study examined the open-source data from Wave 1 of LASI (2017-2018). It is a large-scale national study conducted among the Indian aging population for the assessment of health outcomes, economic status, and social determinants. The International Institute for Population Sciences (IIPS), Mumbai, implemented the study with international collaboration [15]. The information was collected from 72,250 adults aged 45 years or more and their spouses across Indian states and union territories using a “multistage stratified area probability cluster sampling strategy.” The survey results are internationally comparable. Further, the LASI Wave-1 Report contains detailed information on the sampling frame and methodology [15]. The current study focuses on a subset of eligible older Indian adults aged 60 and beyond. The objective of the study is to gain a better understanding of how AYUSH practitioners' services are utilized among older adults, which may provide insights into how these services can be better tailored to the needs of this subgroup population. The study's final sample size was 31,464 (15,098 males and 16,366 females) people aged ≥ 60 years.

Outcome variable

The survey did not have separate questions to inquire about the utilization of AYUSH healthcare providers [15]. Instead, the survey asked respondents a single question about any consultation they had with an AYUSH healthcare provider in the past 12 months. Therefore, the survey does not reveal which specific AYUSH system the respondents used. We coded the consultation with the AYUSH practitioner as a binary (yes/no) outcome variable. It was assessed using the question HC003: “In the past 12 months, have you consulted any AYUSH (Ayurveda /Unani/Siddha/Homeopathy) healthcare provider?”

Explanatory variables

The key explanatory variables were individual, household factors, and self-reported chronic conditions. We categorized age into three groups: 60-69 years old, 70-79 years old, and 80+ years old. We classified the residence status as either urban or rural. Gender was categorized as male or female. The social status categorization included scheduled caste (SC), scheduled tribe (ST), other backward class (OBC), and others. The classification of religions included Hindu, Muslim, Christian, and others. The education level classification included illiterate, less than primary, secondary, higher secondary, and above categories. Working status was categorized as never worked, currently working, or not working. We categorized monthly per capita consumption expenditure (mpce_quintile) into five categories: poorest, poorer, middle, richer, and richest. We coded self-reported chronic disease conditions like diabetes, hypertension, cancer, coronary heart disease (CHD), stroke, arthritis, neurological, cholesterol, and chronic obstructive pulmonary disease (COPD) as binary (yes/no).

Statistical approach

In this study, descriptive statistics used an unweighted number and a weighted percentage to estimate the utilization of services by AYUSH practitioners. In bivariate analysis, the chi-square test was used to see if there were any intergroup differences in the utilization of AYUSH practitioners’ services among older individuals. The association between explanatory variables and AYUSH practitioners' service utilization was established using unadjusted, minimally adjusted, and fully adjusted logistic regression models. The study findings are given as an OR with a 95% confidence interval.

Model 1 provides an estimate of the utilization of AYUSH doctors and is minimally adjusted for residence and gender. Model 2 is fully adjusted for all significant explanatory variables. The RStudio (v. 4.3.2; Posit PBC, Boston, MA) was used to perform statistical analysis [16]. R-script is attached as supplementary material.

## Results

Table 1 represents the general characteristics of participants above 60 years old in India. The total number of participants was 31,464, ranging in age from 60 to 116 years old. Six in 10 participants were in the age group of 60 to 69 years; seven in 10 participants belonged to rural areas; and more than half (53%) belonged to the female gender. The four-fifth participants were either illiterate or less than primary educated. Two-thirds of the participants were either poor or middle class. Three-fourths of the participants were from backward castes. Eight in 10 participants were Hindu, and one in 10 participants was Muslim. Six out of the 10 participants held employment.

**Table 1:** Characteristics of participants aged 60 years and above population *31,464, mpce_quintile: Monthly per Capita Consumption Expenditure, SC: Scheduled Caste, ST: Scheduled tribe, OBC: Other Backward Class; COPD: Chronic Obstructive Pulmonary Disease, CHD: Coronary Heart Disease

Variables	Variables groups	Number** *	Weighted Percentage	95% Confidence Limit
Age (in Years)	60-69	18974	59	(57-60)
	70-79	9101	30	(29-31)
	80 +	3389	11	(11-12)
Residence	Rural	20725	71	(69-72)
	Urban	10739	29	(28-31)
Gender	Male	15098	47	(46-49)
	Female	15366	53	(51-54)
Caste Group	SC	5140	19	(19-20)
	ST	5173	8	(08-09)
	OBC	11886	46	(45-48)
	Other	8218	26	(25-27)
mpce_quintile	Poorest	6484	22	(21-23)
	Poorer	6477	22	(21-23)
	Middle	6416	21	(20-22)
	Richer	6170	19	(18-20)
	Richest	5917	16	(15-17)
Education Level	Illiterate	16889	57	(55-58)
	Less than Primary	7560	23	(22-23)
	Secondary	5560	17	(16-18)
	≥Higher Secondary	1455	4	(04-05)
Religion	Hindu	23037	82	(81-83)
	Muslim	3731	11	(10-12)
	Christian	3150	3	(03-03)
	Other	1546	4	(03-04)
Current Working Status	Never worked	8776	28	(27-29)
	Not working	9307	42	(41-43)
	Working	13373	58	(57-59)

**Figure 1:**
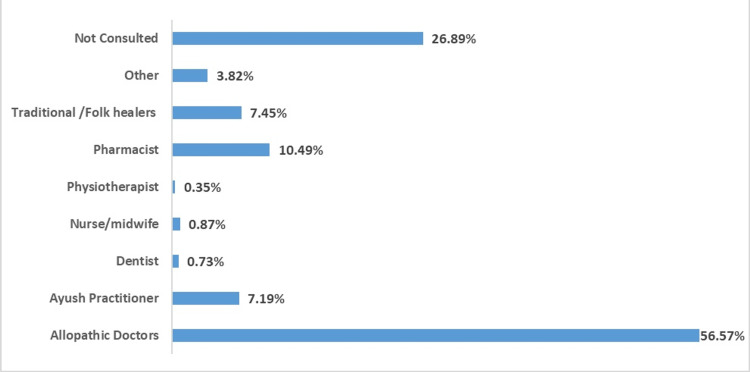
Healthcare worker consultation during the last one preceding the survey The figures represent weighted percentage

Figure 1 depicts the healthcare consultation of older adults with various healthcare workers during the year preceding the survey. One-fourth of older adults did not consult with any healthcare workers during the year preceding the survey. The majority of participants had consulted modern medicine doctors, followed by pharmacists and AYUSH practitioners. Among the consulting older adults, one in 10 opted for an AYUSH practitioner for consultation. Among the older adults consulting AYUSH practitioners, 3.9% and 13.4% (table not given) had consulted at government and private AYUSH hospitals, respectively.

Table 2 represents the socio-economic and health profiles of the older adults consulting AYUSH practitioners. As age increased, the overall consultation with AYUSH practitioners decreased, with no statistical significance. The consultations with AYUSH practitioners were significantly higher in rural areas (1.7 times) and females (1.34 times) than their respective counterparts. There was a significant decline in consultation with AYUSH practitioners with an increase in wealth quintile. A similar but non-significant declining trend was seen with education level. Christians and ST consulted AYUSH practitioners significantly less than their counterparts, while Hindus and SC consulted them the most. Currently working older adults consult AYUSH practitioners more than non-working or never-working older adults, but this difference is not statistically significant. Further, among all the chronic diseases, consultation with an AYUSH practitioner was more common in participants with arthritis, followed by stroke, neurological conditions, and COPD. Consultation for highly prevalent disease conditions such as hypertension, diabetes, etc., was lower.

**Table 2:** Socio-economic and health profile among the older adult consulting AYUSH practitioners mpce_quintile: Monthly per Capita Consumption expenditure, SC: Scheduled Caste, ST: Scheduled tribe, OBC: Other Backward Class; COPD: Chronic Obstructive Pulmonary Disease, CHD: Coronary Heart Disease ^Since unweighted number is less than 25, it has been dropped from the significant test analysis

Explanatory Variable	Category	AYUSH consultation	Weighted Percentage	Chi-square p-value
					Age groups (Years)	60-69	861	7.4	0.967
70-79	419	7							
80+	154	6.7							
Residence	Rural	1073	8.1	<0.001
	Urban	361	4.8	
Gender	Male	640	6.7	0.0125
	Female	794	7.6	
mpce_	Poorest	276	7	0.0266
	Poorer	311	7.9	
	Middle	325	7.8	
	Richer	288	7.3	
	Richest	234	5.6	
Education	Illiterate	778	7.4	0.158
	Less than Primary	170	7.2	
	Secondary	398	7.1	
	≥Higher Secondary	88	5.9	
Religion	Hindu	1185	7.6	<0.001
	Muslim	175	7.1	
	Christian	32	1.2	
	Others	42	3.8	
Caste	SC	285	8	<0.001
	ST	74	4.1	
	OBC	626	6.9	
	Other	415	8.3	
Current	Never worked	375	6.5	0.231
Working	Not working	615	7	
Status	Working	444	8.1	
Chronic Disease Conditions	Hypertension	429	5.6	<0.001
	Diabetes	161	4.2	<0.001
	Cancer^	11	4.2	-
	COPD	122	7	0.175
	CHD	58	3.8	0.1
	Stroke	44	8.1	0.404
	Arthritis	381	9.1	<0.001
	Neurological	41	7.1	0.757
	Cholesterol	50	4.4	0.618

In the logistic regression analysis, it was found that the older adults in the unadjusted model from urban residence, female gender, middle wealth quintile, Christian, other religion, ST, hypertension, diabetes, and arthritis were associated with utilization of AYUSH practitioners’ consultation services (Table 3). In the minimally adjusted model, it was found that the older adults who resided in urban areas utilized AYUSH consultations 38% less than their rural counterparts. Older females were 1.15 times more likely to utilize AYUSH consultation than their male counterparts. Also, Christians and other religious groups were 71% and 44%, respectively, less likely to utilize AYUSH consultation as compared to Hindus. Notably, ST was significantly 67% less likely to visit AYUSH practitioners as compared to SC. Furthermore, older adults without hypertension and diabetes used AYUSH consultations 1.24 and 1.31 times, respectively, compared to those with the disease. However, older adults without arthritis are 41% less likely to utilize an AYUSH consultation. In the fully adjusted model, the effect of explanatory variables is almost similar to that in the minimally adjusted model. Only the effect of the female gender was accentuated in magnitude, whereas the effect of diabetes was partially attenuated.

**Table 3:** Logistic regression estimates access to AYUSH healthcare services utilization by background characteristics among the older adult %: Percentage, Ref.: Reference Category, mpce_quintile: Monthly per capita consumption expenditure, AYUSH: Ayurveda, Yoga & Naturopathy, Unani, Siddha, and Homeopathy, SC: Scheduled Caste, ST: Scheduled tribe, OBC: Other Backward Class *Adjusted for residence and gender. **Adjusted for residence, gender, mpce_quintile, Religion, Caste, Hypertension, Diabetes, Arthritis Significance codes:  0 - ***; 0.001 - **; 0.01 - *

Variables		Category	β (95% CI); Unadjusted	β (95% CI) Model 1; Minimally adjusted	β (95% CI) Model 2; Fully adjusted
Residence	Rural		Ref.	Ref.	Ref.
	Urban		0.64*** (0.56-0.72)	0.62*** (0.51-0.75)	0.62*** (0.55-0.71)
Gender	Male		Ref.	Ref.	Ref.
	Female		1.14 * (1.03-1.27)	1.15 * (1.02-1.31)	1.15**(1.03-1.28)
mpce_quintile	Poorest		Ref.	Ref.	Ref.
	Poorer		1.13 (0.95 -1.33)	1.10 (0.93-1.30)	1.10(0.93-1.30)
	Middle		1.19 * (1.01-1.41)	1.16 (0.98-1.38)	1.16(0.98-1.38)
	Richer		1.79(0.92-1.29)	1.06 (0.89-1.27)	1.06(0.89-1.27)
	Richest		0.92 (0.77-1.10)	0.93 (0.77-1.12)	0.93(0.77-1.12)
Religion	Hindu		Ref.	Ref.	Ref.
	Muslim		0.90 (0.76-1.06)	1.02(0.85-1.21)	1.02(0.85-1.21)
	Christian		0.18 *** (0.12-0.26)	0.29*** (0.18-0.43)	0.29*** (0.18-0.43)
	Others		0.51*** (0.37-0.69)	0.56*** (0.40-0.76)	0.56*** (0.40-0.76)
Caste	SC		Ref.	Ref.	Ref.
	ST		0.24*** (0.18-0.31)	0.33*** (0.25-0.44)	0.33*** (0.25-0.44)
	OBC		0.95 (0.82-1.09)	0.94(0.81-1.09)	0.94(0.81-1.09)
	Other		0.90 (0.77-1.06)	1.02(0.87-1.20)	1.02(0.87-1.20)
Hypertension	Yes		Ref.	Ref.	Ref.
	No		1.27*** (1.13-1.43)	1.24*** (1.09-1.40)	1.24*** (1.09-1.40)
Diabetes	Yes		Ref.	Ref.	Ref.
	No		1.47*** (1.24-1.74)	1.31**(1.09-1.57)	1.31**(1.09-1.57)
Arthritis	Yes		Ref.	Ref.	Ref.
	No		0.58*** (0.51-0.65)	0.59*** (0.52-0.67)	0.59*** (0.52-0.67)

## Discussion

In India, there is a growing demand and governmental effort to mainstream AYUSH, primarily to improve services within the formal healthcare system [2,17]. However, this study reveals that allopathic doctors and pharmacists, not AYUSH practitioners, perform the majority of healthcare consultations for older adults. The majority of AYUSH consultations happened outside the premises of government or private AYUSH hospitals, highlighting the prevalence of non-organized AYUSH practice [18]. The presence of a large number of informal alternative medicine practitioners in both rural and urban India is undocumented [8,12]. Consultation with an AYUSH practitioner indirectly indicates utilization of the AYUSH system of medicine. This study indicates that one in 14 older adults use AYUSH healthcare services. According to a 2014 national survey on health consumption, 6.9% of all patients have sought AYUSH services in the last 15 days [18]. Another study suggested that AYUSH utilization in India was about 7% of outpatient treatment [2]. Similarly, a sub-national survey suggested 14% of patients are in receipt of ISM and homeopathic treatment [14] and a WHO-SAGE survey suggests 11.7% of participants have frequently used “traditional medicine” for healthcare [19]. The WHO-SAGE survey has implicated rural residency and lower socio-economic status with higher use of traditional healer services.

In this study, middle-income quintiles and rural residency have been associated with higher use of AYUSH practitioners’ services. The study by Singh et al. has a contradictory finding that a higher likelihood of ISM and homeopathic medicine was associated with higher income and literacy in the household [14]. People with higher incomes and better reading and writing skills are more likely to use ISM and homeopathy medicines. This suggests that these alternative medicine systems are not just used by people in a certain social or economic group but by a wider range of people from all walks of life. Furthermore, studies in China and Nepal found a positive correlation between higher use of traditional medicine and higher income [20,21]. The studies indicate that cultural beliefs, the availability of practitioners, and personal preferences may also influence the utilization of traditional medicine, in addition to economic factors.

Further, after adjusting for socioeconomic factors, our study finds a significant rural-urban difference; however, a study that used National Sample Survey Organization (NSSO) data (2014) found no such difference in AYUSH healthcare utilization. Similarly, findings from the NSSO data suggest that Muslims are more likely to use AYUSH care [18], but our study reveals higher AYUSH service utilization among Hindu older adults. Ayurveda and Yoga, traditional systems of medicine with their roots in ancient Hindu philosophy and centuries of practice in India, may account for this finding of higher AYUSH service utilization among Hindu older adults. As a result, older adults who practice Hinduism may be more familiar with and trust these medical practices than those who follow other religions or no religion at all. Also, because Ayurveda and Yoga are part of the Hindu religious script and Unani is part of the Muslim religious script, their utilization of AYUSH services is higher than that of Christians, who traditionally prefer modern medicines. However, the LASI data only asks about AYUSH utilization as a whole and does not differentiate between Ayurveda and Unani. Further access to AYUSH services is available to all individuals, regardless of their religion or cultural background.

Higher utilization of AYUSH practitioners’ service among backward castes was noted in our study, similar to the result of an earlier health consumption (NSSO-2014) study [18]. The above differences may be due to the contextual nature of AYUSH service utilization. This study highlights the preference for AYUSH practitioners’ service utilization in certain chronic conditions such as arthritis, stroke, neurological conditions, and COPD in comparison to other chronic conditions such as diabetes, hypertension, cancer, etc. Further study shows that the integration of AYUSH practitioners’ services has the potential to improve the quality of healthcare in India [22]. There is growing recognition of the benefits of combining AYUSH and modern medical practices, particularly in the management of chronic diseases [23]. AYUSH practitioners support chronic condition management like diabetes and hypertension with holistic approaches such as diet advice, yoga, and Ayurveda. They also promote preventive healthcare and mental well-being through practices like meditation, complementing modern healthcare for comprehensive care [24-27]. In recent years, several AYUSH-related research projects have been conducted, but many have unclear objectives and methodologies that need formal training [28]. The MoA has specified the purpose and target group of these digital initiatives, categorizing them under Health Information System, Research Database/Library, Academic, and IEC. The MoA's digital initiatives are playing a key role in reforming traditional medicine systems and improving AYUSH healthcare services' education, quality of research, and accessibility.

The study's strength lies in its use of nationally representative data from the LASI, offering insights into AYUSH service utilization trends among older adults over time. The study adds to the limited evidence available on AYUSH consultation or service utilization.

However, it is crucial to acknowledge the limitations of secondary data, biases such as recall biases in self-reported health data, and the inherent constraints of observational research for establishing causal relationships. Consideration of AYUSH which includes four different types of ISMs with differential client profiles, may limit our understanding of utilization of individual ISM services.

## Conclusions

The study highlights that despite governmental efforts to mainstream AYUSH into India’s formal healthcare system, allopathic medicine remains dominant for older adults. Older adults prefer to use AYUSH services for chronic conditions like arthritis, stroke, neurological conditions, and COPD. Integrating AYUSH services into mainstream healthcare can potentially enhance chronic disease management through holistic approaches. The AYUSH, which is popular among female and rural residents, may help improve essential care coverage for the underprivileged. The practice of non-organized AYUSH is widespread and requires regulation. Given that AYUSH is a combination of four ISM, future waves of LASI surveys should consider collecting data for each system of medicine to gain a comprehensive understanding of individual ISM service utilization.

## Appendices

R-script used for the study:

library(epicalc)

library(epitools)

library(gmodels)

library(gtools)

library(pspline)

library(epiR)

library(BiasedUrn)

library(gdata)

library(Gmisc)

library(Rcpp)

library(htmlTable)

library(MASS)

library(foreign)

library(survival)

library(nnet)

library(descr)

use("D:\\Research\\LASI_Ayush\\AYUSH.dta")

AYUSH=.data

attach(AYUSH)

View(AYUSH)

##outcome

tab1(hc003)  #In the past 12 months, have you consulted any AYUSH (Ayurveda /unani/ siddha /homeopathy) health care provider?

# Create the binary outcome variable for AYUSH consultation

AYUSH$AYUSH_consulted <- ifelse(df$hc003_b == 1, "Yes", "No")

#summary of the new variable

table(AYUSH$AYUSH_consulted)

# Descriptive statistics

descriptive_stats <- table(AYUSH$AYUSH_consulted)

prop.table(descriptive_stats)

# Creating a table with general characteristics

tab1(residence)

tab1(dm008) #age

age<-dm008

tab1(dm003)#gender

gender<-dm003

tab1(dm021)#marital status

marital_status<-dm021

tab1(living_arrangements)#living arrangement

tab1(dm010)#religion

religion<-dm010

tab1(dm013)#caste

caste<-dm013

tab1(dm008)#education

education<-dm008

tab1(we004)#work status

work_status<-we004

tab1(mpce_quintile)#mpcequantiles

tab1(ht002)

Hypertension<-ht002 ##Hypertension

tab1(ht003)

Diabetes<-ht003##Diabetes

tab1(ht004)

Cancer<-ht004#Cancer

tab1(ht005)

COPD<-ht005 #COPD

tab1(ht006)

CHD<-ht006 #CHD

tab1(ht007)

Stroke<-ht007 #Stroke

tab1(ht008)

Arthritis<-ht008##Arthritis

tab1(ht009)

Neurological<-ht009#Neurological

tab1(ht010)

Cholesterol<-ht010 #Cholesterol

general_char <- CreateTableOne(vars = c("age", "residence", "gender", "caste", "religion", "education","marital_status", "work_status", "mpce_quintile","Hypertension", "Diabetes", "Cancer","COPD", "CHD", "Stroke", "Arthritis","Neurological","Cholesterol"),

                               data = AYUSH, factorVars = c("age", "residence", "gender", "caste", "religion", "education","marital_status", "work_status", "mpce_quintile","Hypertension", "Diabetes", "Cancer","COPD", "CHD", "Stroke", "Arthritis","Neurological","Cholesterol"))

print(general_char, showAllLevels = TRUE)

# Bivariate analysis using chi-square tests

# residence

CrossTable(AYUSH$residence, AYUSH$AYUSH_consulted, prop.r = TRUE, chisq = TRUE)

CrossTable(AYUSH$age, AYUSH$AYUSH_consulted, prop.r = TRUE, chisq = TRUE)

CrossTable(AYUSH$gender, AYUSH$AYUSH_consulted, prop.r = TRUE, chisq = TRUE)

CrossTable(AYUSH$marital_status, AYUSH$AYUSH_consulted, prop.r = TRUE, chisq = TRUE)

CrossTable(AYUSH$living_arrangements, AYUSH$AYUSH_consulted, prop.r = TRUE, chisq = TRUE)

CrossTable(AYUSH$religion, AYUSH$AYUSH_consulted, prop.r = TRUE, chisq = TRUE)

CrossTable(AYUSH$caste, AYUSH$AYUSH_consulted, prop.r = TRUE, chisq = TRUE)

CrossTable(AYUSH$education, AYUSH$AYUSH_consulted, prop.r = TRUE, chisq = TRUE)

CrossTable(AYUSH$work_status, AYUSH$AYUSH_consulted, prop.r = TRUE, chisq = TRUE)

CrossTable(AYUSH$mpce_quintile, AYUSH$AYUSH_consulted, prop.r = TRUE, chisq = TRUE)

CrossTable(AYUSH$Hypertension, AYUSH$AYUSH_consulted, prop.r = TRUE, chisq = TRUE)

CrossTable(AYUSH$Diabetes, AYUSH$AYUSH_consulted, prop.r = TRUE, chisq = TRUE)

CrossTable(AYUSH$Cancer, AYUSH$AYUSH_consulted, prop.r = TRUE, chisq = TRUE)

CrossTable(AYUSH$COPD, AYUSH$AYUSH_consulted, prop.r = TRUE, chisq = TRUE)

CrossTable(AYUSH$CHD, AYUSH$AYUSH_consulted, prop.r = TRUE, chisq = TRUE)

CrossTable(AYUSH$Stroke, AYUSH$AYUSH_consulted, prop.r = TRUE, chisq = TRUE)

CrossTable(AYUSH$Arthritis, AYUSH$AYUSH_consulted, prop.r = TRUE, chisq = TRUE)

CrossTable(AYUSH$Neurological, AYUSH$AYUSH_consulted, prop.r = TRUE, chisq = TRUE)

CrossTable(AYUSH$Cholesterol, AYUSH$AYUSH_consulted, prop.r = TRUE, chisq = TRUE)

# Logistic Regression Models

# Unadjusted model

model_unadjusted <- glm(AYUSH_consulted ~ residence + gender + age + caste + religion + education + work_status + mpce_quintile + Hypertension + Diabetes + Arthritis, data = AYUSH, family = binomial)

summary(model_unadjusted)

exp(cbind(OR = coef(model_unadjusted), confint(model_unadjusted)))

# Minimally adjusted model (adjusted for residence and gender)

model_minimally_adjusted <- glm(AYUSH_consulted ~ residence + gender, data = AYUSH, family = binomial)

summary(model_minimally_adjusted)

exp(cbind(OR = coef(model_minimally_adjusted), confint(model_minimally_adjusted)))

# Fully adjusted model (adjusted for all significant variables)

model_fully_adjusted <- glm(AYUSH_consulted ~ residence + gender + age + caste + religion + education + work_status + mpce_quintile + Hypertension + Diabetes + Arthritis, data = AYUSH, family = binomial)

summary(model_fully_adjusted)

exp(cbind(OR = coef(model_fully_adjusted), confint(model_fully_adjusted)))

## References

[1] Samal J (2015). Role of AYUSH workforce, therapeutics, and principles in health care delivery with special reference to National Rural Health Mission. Ayu.

[2] Ravishankar B, Shukla VJ (2007). Indian systems of medicine: a brief profile. Afr J Tradit Complement Altern Med.

[3] Amrith S (2007). Political Culture of Health in India: A Historical Perspective. Econ Polit Weekly.

[4] Mushtaq MU (2009). Public health in British India: a brief account of the history of medical services and disease prevention in colonial India. Indian J Community Med.

[5] Srinivasan P (1995). National health policy for traditional medicine in India. World Health Forum.

[6] (2024). Meeting people’s health needs in rural areas: framework for Implementation, 2005-2012. National Rural Health Mission.

[7] Gupta PK, Karthik KP, Sahu R, Shrikrishna R, Mahapatra A (2023). Public health initiatives and Ayush: projects to policy. Int J Ayurveda Res.

[8] Ashtekar S, Mankad D (2001). Who cares? Rural health practitioners in Maharashtra. Econ Polit Weekly.

[9] Ramesh A, Hyma B (1981). Traditional Indian medicine in practice in an Indian metropolitan city. Soc Sci Med Med Geogr.

[10] Neumann AK, Bhatia JC, Andrews S, Murphy AK (1971). Role of the indigenous medicine practitioner in two areas of India. Report of a study. Social Sci Med.

[11] Pengpid S, Peltzer K (2021). Utilization of complementary and traditional medicine practitioners among middle-aged and older adults in India: results of a national survey in 2017-2018. BMC Complement Med Ther.

[12] Lakshmi JK, Nambiar D, Narayan V, Sathyanarayana TN, Porter J, Sheikh K (2015). Cultural consonance, constructions of science and co-existence: a review of the integration of traditional, complementary and alternative medicine in low- and middle-income countries. Health Policy Plan.

[13] Oyebode O, Kandala NB, Chilton PJ, Lilford RJ (2016). Use of traditional medicine in middle-income countries: a WHO-SAGE study. Health Policy Plan.

[14] Jadhav U, Mukherjee K, Thakur H (2013). Usage of complementary and alternative medicine among severe hemophilia A patients in India. J Evid Based Complementary Altern Med.

[15] (2024). Longitudinal Ageing Study in India (LASI). https://www.iipsindia.ac.in/lasi.

[16] The R Foundation (2022). The R Foundation. https://www.r-project.org/.

[17] Sharma P, Khan IA (2022). Reviving Ayurveda as a component of AYUSH. Int J Cur Res Rev.

[18] Rudra S, Kalra A, Kumar A, Joe W (2017). Utilization of alternative systems of medicine as health care services in India: evidence on AYUSH care from NSS 2014. PLoS One.

[19] Febriyanti RM, Saefullah K, Susanti RD, Lestari K (2024). Knowledge, attitude, and utilization of traditional medicine within the plural medical system in West Java, Indonesia. BMC Complement Med Ther.

[20] Thorsen RS, Pouliot M (2016). Traditional medicine for the rich and knowledgeable: challenging assumptions about treatment-seeking behaviour in rural and peri-urban Nepal. Health Policy Plan.

[21] Elwell-Sutton TM, Jiang CQ, Zhang WS, Cheng KK, Lam TH, Leung GM, Schooling CM (2013). Inequality and inequity in access to health care and treatment for chronic conditions in China: the Guangzhou Biobank Cohort Study. Health Policy Plan.

[22] Chandra S (2012). Status of Indian medicine and folk healing: with a focus on integration of AYUSH medical systems in healthcare delivery. Ayu.

[23] Chandra S, Patwardhan K (2018). Allopathic, AYUSH and informal medical practitioners in rural India - a prescription for change. J Ayurveda Integr Med.

[24] Sharma K, Basu-Ray I, Sayal N (2022). Yoga as a preventive intervention for cardiovascular diseases and associated comorbidities: open-label single arm study. Front Public Health.

[25] Patwardhan B, Sarwal R (2021). Significance of AYUSH: India's first line of defence against COVID-19. J Ayurveda Integr Med.

[26] Gautam S, Gautam A, Chhetri S, Bhattarai U (2022). Immunity against COVID-19: potential role of Ayush Kwath. J Ayurveda Integr Med.

[27] Malhotra V, Sampath A, Javed D (2022). Yoga as an escape from depreciating mental health due to COVID 19: a qualitative study analyzing the factors associated with mental status based on the experiences of geriatric population’s participation in an online program during COVID 19 lockdown in India. Int J Yoga.

[28] Tillu G (2019). AYUSH and meta-research. J Ayurveda Integr Med.

